# Raman tensor elements of *β*-Ga_2_O_3_

**DOI:** 10.1038/srep35964

**Published:** 2016-11-03

**Authors:** Christian Kranert, Chris Sturm, Rüdiger Schmidt-Grund, Marius Grundmann

**Affiliations:** 1 Universität Leipzig, Institut für Experimentelle Physik II, Abteilung Halbleiterphysik, Linnéstraβe 5, 04103 Leipzig, Germany

## Abstract

The Raman spectrum and particularly the Raman scattering intensities of monoclinic *β*-Ga_2_O_3_ are investigated by experiment and theory. The low symmetry of *β*-Ga_2_O_3_ results in a complex dependence of the Raman intensity for the individual phonon modes on the scattering geometry which is additionally affected by birefringence. We measured the Raman spectra in dependence on the polarization direction for backscattering on three crystallographic planes of *β*-Ga_2_O_3_ and modelled these dependencies using a modified Raman tensor formalism which takes birefringence into account. The spectral position of all 15 Raman active phonon modes and the Raman tensor elements of 13 modes were determined and are compared to results from ab-initio calculations.

Gallium oxide in its stable *β*-modification is a semiconductor with a very wide bandgap of approximately 4.8 eV^1^,^2^. This makes this material an interesting candidate as active medium in deep UV optoelectronics. Further, the wide bandgap suggests a theoretically larger breakdown field than for e.g. Si or SiC indicating a potential application of *β*-Ga_2_O_3_ in high power electronics^3^.

For the use of the material in such applications, the knowledge of its fundamental properties is vital. These include the phonon energies as obtained by Raman spectroscopy which give access to sample properties like strain. In several experimental^4^,^5^,^6^ and theoretical^4^,^5^,^7^ studies, the energies of the Raman-active phonon modes have been reported. We briefly review these results and compare them to our own findings.

The main focus of the present paper is on the information that can be obtained from the Raman scattering intensities. Since *β*-Ga_2_O_3_ has a monoclinic crystal structure, its properties are strongly anisotropic. The investigation of Raman intensities provides access to the orientation of a particular sample via the selection rules as well as via the dependence of the Raman scattering intensity for the individual phonon modes on the polarization relative to the crystal orientation. However, the well-known relation between scattering intensity *I* and scattering geometry


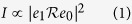


with the polarizations of the incident and scattered light *e*_1_ and *e*_0_ at the point of the scattering event cannot be directly applied to experiments on anisotropic crystals. Owing to birefringence, the polarization of the radiation within the crystal, where the scattering event occurs, is in general elliptical and different from the incident and detected polarizations *e*_i_ and *e*_s_ determined by the experimental setup. Further, this effect is depth-dependent such that it was considered to be “pointless”^8^ to analyse the Raman intensities for polarizations which are not parallel to the principal axes of the dielectric indicatrix. Therefore, only Raman intensities for the polarization configurations parallel to the principal axes were reported for *β*-Ga_2_O_3_ so far^4^.

This experimental limitation causes severe limitations for gaining information on the Raman tensor. In application, it is of course desirable to calculate the scattering intensity for any orientation of the crystal in order to determine the orientation of a given sample. The restriction to polarization directions parallel to the principal axes further prevents the determination of the signs of the Raman tensor elements since only intensities are measured. Fortunately, for backscattering with sufficiently large scattering depth range, which is typically the case for bulk samples, the depth dependence vanishes and the scattering intensities can be described using a modified Raman tensor formalism^9^. Here, we apply this formalism to model the Raman intensities for various scattering geometries and this way obtain the individual Raman tensor elements including their sign for most Raman active phonon modes of *β*-Ga_2_O_3_.

## Results and Discussion

### Phonon energies

The stable form of gallia under ambient conditions is monoclinic *β*-Ga_2_O_3_ which belongs to the space group *C*2/*m* in international and 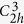 in Schönflies notation. The [010]-direction is perpendicular to [100] and [001] which confine an angle *β* = 103.7°. Because the [010]-direction is perpendicular to the other two crystal axes lying in the (010)-plane, we choose 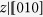 in the following. The assignment of the in-plane coordinates is discussed below.

The primitive unit cell of *β*-Ga_2_O_3_ consists of 10 atoms which results in 30 phonon modes of which 27 are optical modes. At the Γ-point, these belong to the irreducible representation^4^


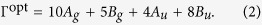


The modes with *A*_*g*_ and *B*_*g*_ symmetry are Raman active, while those with odd parity (index *u*) are infrared active. Under non-resonant conditions, the Raman tensors for the Raman-active phonon modes have the form^10^


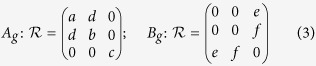


with real tensor elements for excitation in the transparency regime.

**Figure 1:**
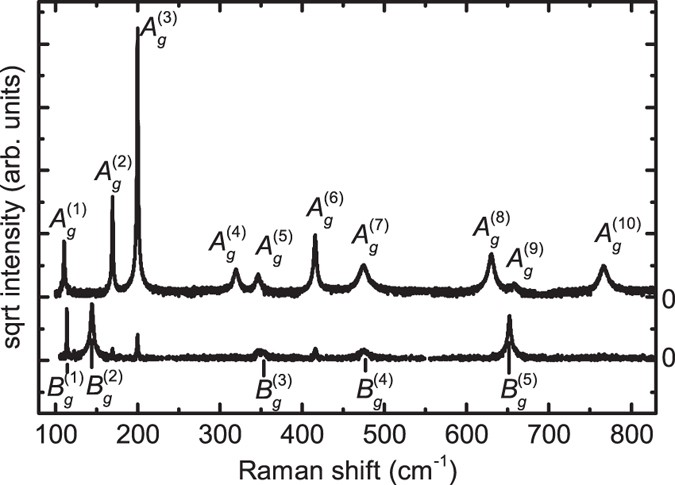
Experimental Raman spectra of two *β*-Ga_2_O_3_ single crystals excited at *λ* = 532 nm. Top curve: (010)-oriented crystal, scattering geometry *z(yy*)*z*, bottom curve: 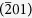-oriented crystal, scattering geometry *z*′(*x*′*y*′)*z*′, where 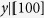, 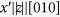, 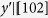, and 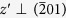.

The form of the Raman tensors induce selection rules, that means that the Raman modes of either symmetry can be extinguished for certain polarization configurations. This allows to distinguish between the two phonon mode symmetries as in the experimental Raman spectra shown in Fig. 1. For excitation on the (010)-plane, the *B*_*g*_ modes are forbidden and the *A*_*g*_ modes are allowed for all polarization configurations. For an excitation on a surface perpendicular to that, both types of modes can be allowed, depending on the polarization configuration. In the cross polarized configuration shown in the bottom curve of Fig. 1, only the *B*_*g*_ modes are allowed. The imperfect extinction of the Raman lines corresponding to the *A*_*g*_ modes results mainly from the limited degree of linear polarization of the laser source and from slight depolarization caused by the experimental setup, particularly induced by the beam splitter and the edge filter. The actual extinction ratio of the used setup is approximately 1:50.

The spectral positions of the individual Raman modes were obtained by modelling their spectral line shape using Lorentzian functions. These experimental results are summarized in Table 1 and compared to our results from ab-initio calculations as well as to values from the literature. A very good agreement between theory and experiment is obvious. The comparison between the experimental results alone shows a good agreement between our results and those of refs 4 and 5 with only small deviations for some phonon modes. We obtain identical results within a margin of error of 0.2 cm^−1^ when using an alternative excitation wavelength (*λ* = 325 nm instead of *λ* = 532 nm used for the results presented here) or sintered powder samples.

The comparison of the theoretical results shows a better agreement for our calculations with those of Dohy *et al.*^4^ than with those obtained using the VASP^5^ and abinit code^7^, which both use plane-wave basis sets as opposed to the Gaussian-type orbital basis sets used by CRYSTAL14 utilized by us. The agreement of the calculations of Dohy *et al.*^4^ with experimental results is similarly good as for our calculations, which is particularly remarkable regarding the very limited computing power available at the time of their work.

### Raman tensor elements

#### Theoretical background

Raman scattering intensities were modelled using the formalism introduced by us in a preceding publication^9^. In its general form, the dependence of the Raman intensity on the polarization configuration for normal-incidence backscattering on a certain surface is given by


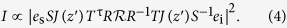


Here, *e*_i_ and *e*_s_ are the normalized polarization vectors of the incident and detected field, respectively. *S* is the rotational matrix transforming the in-plane coordinates of the laboratory system into the in-plane coordinate system (*x*′, *y*′) spanned by the fast and the slow axis of the crystal. Further, *J(z*′) is the Jones matrix for light propagating along the surface normal *z*′. The transformation matrix


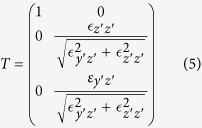


transforms the external polarization, which is pinned to the *x*′-*y*′-plane, to the allowed internal polarizations, which may also exhibit an out-of-plane (*z*′) component if none of the principal axes is parallel to *z*′. The rotational matrix *R* rotates the Raman tensor 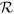, which is defined in the system of principal axes of the indicatrix *x*, *y* and *z*, into the coordinate system *x*′, *y*′, *z*′ determined by the sample orientation.

Birefringence further causes different reflection coefficients at the surface. For normal incidence and vanishing absorption, it is described by the well-known equation


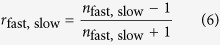


for the fast and slow axis, respectively. In order to properly model our experimental intensities, we took this into account by inserting the diagonal matrix *ρ* = diag((1 − *r*_*x*′*x*′_)/(1 − *r*_*y*′*y*′_), 1) between *S* and *J*. Since no absolute intensities were measured, only the relative reflectivity was considered.

Even though we assume strictly normal incidence, the solid angle inside the crystal, from which light is collected by the focusing optics, differs in dependence on the refractive index. The solid angle is calculated as


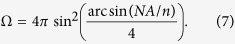


Similarly to the influence of reflection, we introduce a diagonal matrix 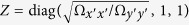 as correction. More precisely, the light cone from which light is collected is in general elliptically shaped due to oblique rays. This even smaller correction is neglected here. Equation (4) then reads


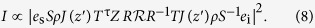


Regarding the form of equation (8), 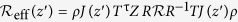 acts as a two-dimensional, depth-dependent effective Raman tensor. We have shown that for integration over a sufficient depth range, the depth dependence vanishes and the scattering intensity can be expressed as^9^


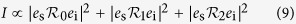


with the three components


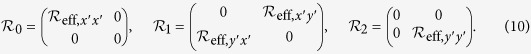


Our previous calculations show that this approximation can be applied for typical experimental conditions present for the investigation of bulk material^9^. We verified the validity of this approximation for the present studies by measuring the actual depth intensity profile of our Raman setup and taking into account the birefringence for the crystal cuts of *β*-Ga_2_O_3_ measured by us. The results can be found in the supplementary material.

The scattering intensity further depends on the phonon frequency *ω*_P_ and incident photon frequency *ω*_i_, i.e. explicitly 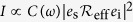 with^10^


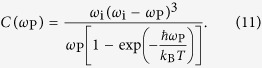


In order to obtain Raman tensor elements which can be compared between the individual phonon modes, this prefactor must be taken into account.

#### Determination of the Raman tensor elements

**Figure 2:**
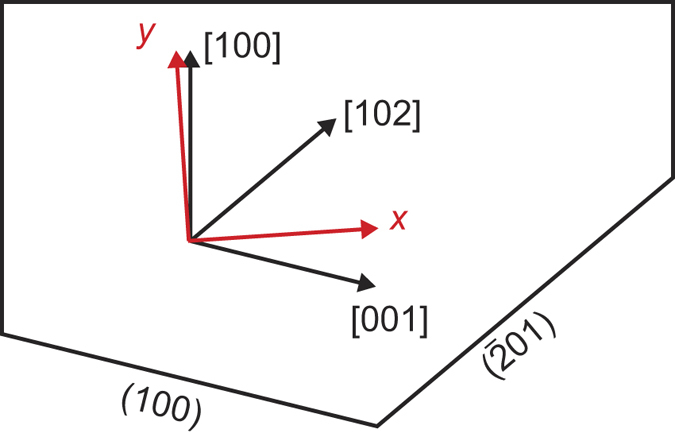
Relation between the crystallographic axes and the coordinate system used here. The [010]-direction points out of the drawing plane, the depicted directions are in the [010]-plane. The crystal cuts yielding (100)- and 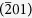-orientation are shown on the bottom. The angle between *y* and [100] is 3.8°, that between *x* and [001] is *θ*_(100)_ = −17.6° and that between *x* and [102] is 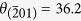
°.

Within the static approximation, i.e. for off-resonant excitation, the Raman tensor represents a modulation of the dielectric tensor. Therefore, it is defined in the coordinate system of the principal axes of the dielectric indicatrix which need to be known in order to analyze the Raman tensor. For the monoclinic system of *β*-Ga_2_O_3_, one of these axes is parallel to the crystallographic [010]-direction. In order to determine the other two principal axes of the indicatrix, we used the dielectric tensor at the excitation wavelength derived from ellipsometry measurements^11^


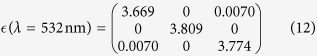


given for a *crystal* coordinate system with 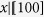, 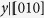 and *z* chosen accordingly as used in ref. 11. From the eigenvectors of this tensor, one finds that the principal axes are tilted by an angle of 3.8° relative to the coordinate system defined above. In the following, the *dielectric* coordinate system as indicated in red in Fig. 2 is used as coordinate system for which the Raman tensors take the form (3). Its axes are parallel to the principal axes of the dielectric indicatrix and the *z*-axis points in the [010]-direction.

**Figure 3:**
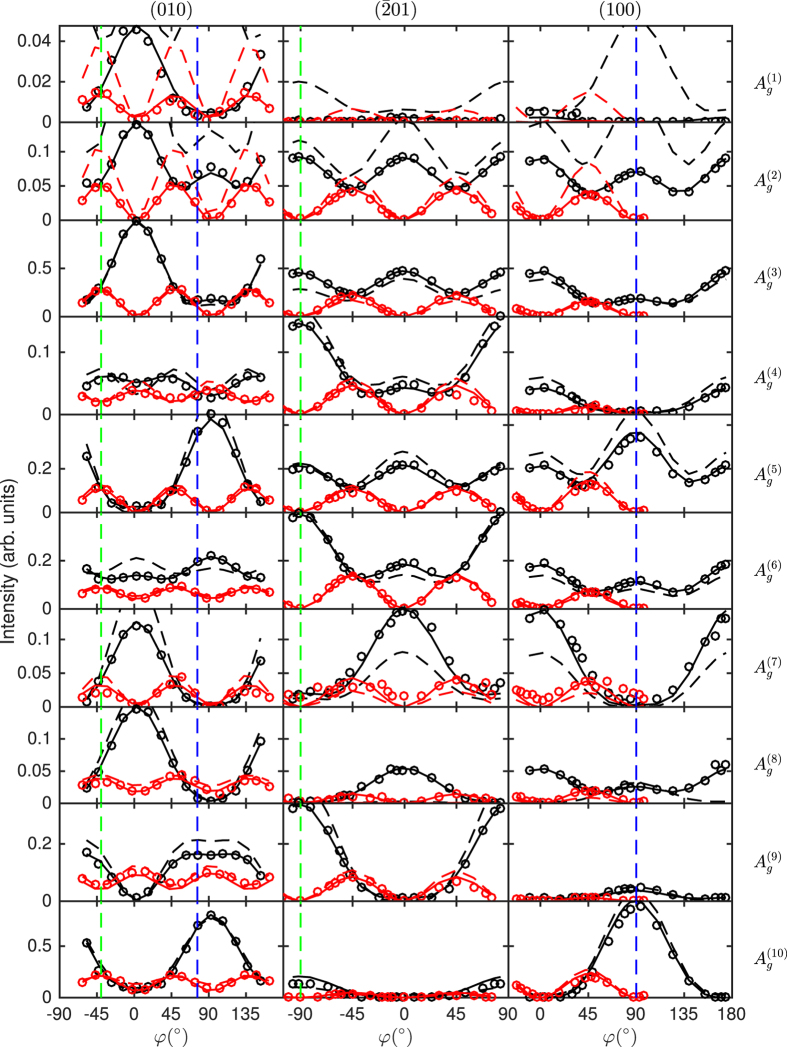
Experimental Raman scattering intensities (circles), model fits (solid lines) and modelled intensities from ab-intio-calculated tensor elements (dashed lines) of the phonon modes with *A*_*g*_-symmetry of *β*-Ga_2_O_3_ for parallel (black) and cross polarization (red) in dependence on the direction of the (scattered) polarization *φ*. The orientation of the excited surface is indicated on top of the spectra and the phonon modes right to the spectra. An intensity of 1 means the overall maximum intensity which is observed for 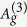. The intensity range is the same for all plots of a single phonon mode. For the (010)-orientation, *φ* = 0° is set such that it coincides with the [100]-direction, for the other two orientations *φ* = 0° corresponds to the [010]-direction. The dashed green and blue lines indicate the [102]- and [001]-direction, respectively.

The three different crystal cuts investigated here are indicated in Fig. 2. We introduce a *sample* coordinate system, indicated by a prime, such that the surface normal is parallel to *z*′. The in-plane coordinates are determined by the orientation of the slow and the fast axis and the transformation between sample and dielectric coordinate system is given by the rotation matrix *R* as in equation (8). For the (010)-plane, *z* = *z*′ such that no transformation is necessary and sample and dielectric coordinate system are identical. For the other two crystal cuts, one of the allowed polarization directions is parallel to the *z*-axis. With *z*′ being the direction of light propagation and *x*′ set parallel to [010], *y*′ is obtained by rotating *x* by an angle *θ* around *z*. Since the propagation direction is not parallel to a principal axis, the polarization component perpendicular to that is slightly tilted against the surface requiring to use the elements of the dielectric tensor to determine the matrix *T* in equation (8). Further, this polarization direction is not parallel to any of the principal axes. Thus, the scattering intensity for this direction results from a linear combination of Raman tensor elements, allowing to access their sign. Since no sample with the [010]-direction tilted against the surface was available for our investigations, this was only possible for the tensor elements *a*, *b* and *d*. Further, the differences in reflectivity caused by birefringence were taken into account using the reflectivity ratio *r*_slow_/*r*_fast_ between the slow and the fast axis for the amplitude of the radiation. The parameters obtained from the dielectric tensor for the three surfaces required for modeling are summarized in Table 2. As discussed above, equation (9) is expected to approximately hold for all investigated orientations. This is in agreement to the experiment judging from the line shapes in Fig. 3.

**Figure 4:**
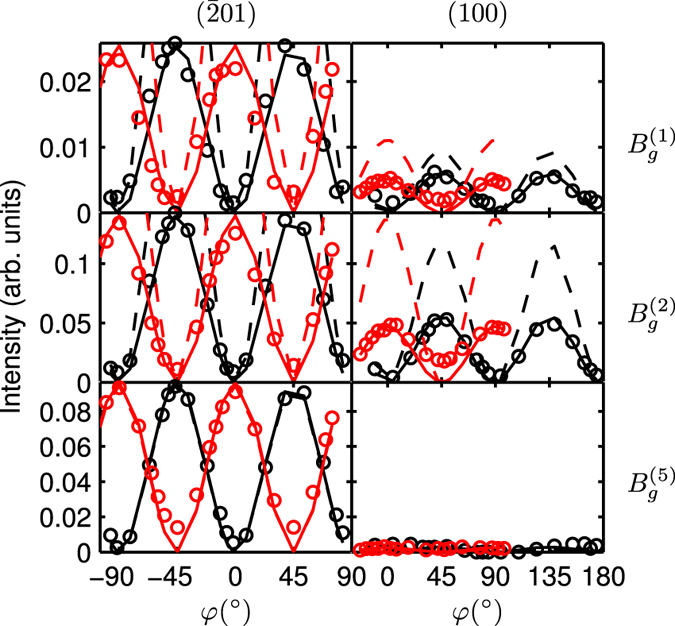
Same as Fig. 3 for the phonon modes with *B*_*g*_ symmetry as indicated. The intensity of 1 is defined as in Fig. 3.

We carried out Raman measurements in backscattering geometry on the three mentioned surfaces with the polarization direction rotated by the angle *φ* relative to the crystal. The intensities for the individual phonon modes were obtained as the area of Lorentzian functions used to model the line shape of the respective Raman peaks. Plotting these intensities over the angle *φ* representing the direction of the polarization relative to the crystal yields graphs as in Figs 3 and 4. In order to assure comparability, the intensities were normalized to the maximum intensity of the prominent peak of the 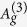 phonon mode at 200 cm^−1^.

We determined the polarization-dependent Raman intensities for all ten phonon modes with *A*_*g*_ symmetry and successfully modelled these dependencies with great agreement using the formalism for anisotropic crystals^9^ as depicted in Fig. 3. Only the four Raman tensor elements *a*, *b*, *c* and *d* were used as free parameter to fit the six polarization dependencies for each phonon mode. When necessary, the intensities for a measurement set were adjusted using an additional scaling factor which is constant for all phonon modes. Similarly, a possible misalignment of the sample was adjusted using a constant angular offset (<3°) for all modes. The required parameters resulting from the dielectric tensor were taken from the ellipsometry results as listed in Table 2.

The effect of birefringence can be clearly seen by two effects in the plots for the *A*_*g*_ modes in Fig. 3: First, without mode conversion due to birefringence, the intensity for excitation on the 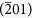
- and (100)-planes is expected to either vary between a maximum and minimum at *φ* = 0° and *φ* = 90°, respectively, or to have an intensity of zero in between. Second, the intensity for the polarization parallel to the [102]-direction is different for excitation on the 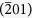
- and (010)-planes. The same is true for the polarization parallel to the [001]-direction on the (100)- and (010)-planes (see dashed lines in Fig. 3). This effect cannot be expected from equation (1), but results from the fact that light with polarizations in these directions is split into two waves propagating in the crystal with different velocities, while this does not occur for the other two planes. In the latter case, additionally the tilting of the polarization relative to the surface must be considered, which is however a much smaller correction.

We modelled the observed intensity dependencies for each phonon mode individually, i.e. we neglected the phonon energy-dependent prefactor (11). The Raman tensor elements were then obtained from the fitting parameters by dividing by 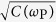. They are summarized in Table 3. The values are normalized to the largest one which is the *a* tensor element of the 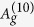 mode. Since the sign of the Raman tensor element *c* could not be determined, its values are all given as positive. As discussed below, it is reasonable to adopt the signs from the computed results. The signs of the other tensor elements are given with respect to *a*, i.e. *a* is set as positive for all phonon modes. Please note that the Raman tensor elements enter quadratically in the scattering intensity.

Only three out of the five phonon modes with *B*_*g*_ symmetry are shown in Fig. 4. The other two modes are too weak and for most scattering geometries superimposed by the spectrally close 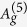 and 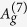 modes. Further, modes with this symmetry cannot be observed for excitation on the (010)-plane owing to the selection rules. Therefore, they are only shown for the other two orientations in Fig. 4. From only these two orientations, the signs of the Raman tensor elements cannot be determined unambiguously, i.e. the polarization dependencies could be equivalently modelled using different Raman tensor elements with opposite signs. Again, the agreement between experiment and theory indicates that the option for the experimental values given in the table is likely to be the correct one.

Theoretical values for the Raman tensor elements were obtained using the Raman intensity option implemented in CRYSTAL14 based on the coupled perturbed Hartree-Fock/Kohn-Sham (CPHF/KS) method. Since the output is restricted to intensities, it does not give access to the signs of the tensor elements. In order to obtain this property, we calculated the first-order dielectric tensor using the CPHF/KS method for the equilibrium crystal configuration and for the crystal with atomic displacement according to the vibrational movement for the individual phonon modes which was taken from the frequency calculation output. The comparison of the values in Tables 3 and 4 shows a good agreement between theory and experiment. This can also be seen from the plot of the modelled scattering intensity in Figs 3 and 4. For these, the Raman intensities were calculated setting the experimental conditions (temperature and excitation wavelength) in agreement to our experimental setup which is equivalent to multiplying with the prefactor *C(ω*_P_) from equation (11). In particular, the general line shape is very well reproduced for most phonon modes, with distinct exceptions particularly for the 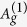 and 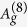 mode. Further, the intensity of the low energy modes 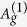, 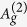, 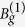 and 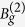 is strongly overestimated. Except for some deviations in the magnitude of individual Raman tensor elements, the agreement for the other phonon modes is very good.

In order to compare our results to the absolute intensities reported by Dohy *et al.*^4^ for polarization directions parallel to principal axes of the indicatrix, the Raman tensor elements must be squared and multiplied with *C(ω*_P_) from equation (11). When doing so, almost identical values with only minor deviations to these results^4^ are obtained. However, for the results to agree one has to assume that Dohy *et al.* confused the *x*- and *y*-axis defined by them similar to our definition. That means that their data for “XX” polarization corresponds to a polarization parallel to the *y*-axis of our dielectric coordinate system, which is close to the [100]-direction.

The remarkably good agreement between the plainly theoretical data combining ab-initio calculations and the model for anisotropic crystals^9^ opens an additional way to identify phonon modes beyond the simple assignment based on selection rules which can be ambiguous under certain conditions. By the comparison between the polarization dependencies from theory and experiment, not only the phonon symmetry, but the actual vibrational mode can be assigned. Usually, this was done solely based on the spectral position which might yield erroneous results. In order to make use of the polarization dependence of the Raman intensity for any crystallographic orientation, it renders necessary to not only investigate Raman intensities, but the actual Raman tensor elements, particularly including their sign. The full Raman tensor also in general enables the determination of the crystallographic orientation of a given sample from the relative Raman intensities of the individual phonon modes. However, the latter task requires a high accuracy of the Raman tensor elements which cannot (yet) be provided by ab-initio calculations as shown by our results. For this problem, experimental Raman tensor elements, as determined here for *β*-Ga_2_O_3_, are necessary. Nevertheless, due to the good agreement, it seems reasonable for example to assign the sign of the Raman tensor element *c* of the *A*_*g*_ phonon modes based on the ab-initio calculations.

## Conclusions

We studied the Raman spectrum of *β*-Ga_2_O_3_ with particular focus on the Raman intensity. We modeled the dependency of the intensity on the scattering configuration for most phonon modes of *β*-Ga_2_O_3_, successfully applying the model for anisotropic crystals. From that, we obtained the experimental Raman tensor elements for these modes and found a good agreement with results from ab-initio calculations. The experimental accessibility of the signs of the Raman tensor element and their impact on the actual scattering intensities, which can be well-modelled, strongly suggest not only to investigate the absolute Raman intensities, but the tensor elements themselves by both experiment and theoretical calculations.

## Methods

We used three different cuts of *β*-Ga_2_O_3_ single crystals as samples: commercial, (010)- and 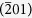-oriented samples from Tamura Corporation and (100)-oriented samples from Leibniz-Institut für Kristallzüchtung (IKZ) Berlin. The crystals are free of twins which we verified by means of X-ray diffraction. Samples from both sources show identical Raman shifts within the margin of error. The same is true for a comparison between unintentionally doped and Sn-doped samples from Tamura. Thus, these specifics of the samples were neglected.

Raman scattering was excited using a diode-pumped solid state laser emitting at *λ* = 532 nm. The incident light was focused on the sample by a microscope objective with a magnification of 50x and a numerical aperture of *NA* = 0.42; the scattered light was collected by the same objective (backscattering geometry). As shown in the supplementary material, the influence of oblique rays due to the focusing aperture can be neglected. The sample was placed with its polished surface perpendicular to the direction of light propagation. An achromatic *λ*/2 waveplate was placed between beam splitter and objective and rotated by an angle of *φ*/2 in order to rotate the polarization of both the incident and detected radiation by an angle of *φ* relative to the sample. The Glan-Thompson polariser used as analyser was kept fix. Another *λ*/2 waveplate was applied in front the beam splitter either at 0° or 45° to select between parallel polarization or cross polarization. The spectrum was recorded using a double spectrometer with 2 × 1 m focal length, gratings with 2400 lines per mm and equipped with a liquid nitrogen-cooled charged-coupled device camera with a pixel pitch of 13.5 μm. The slit width was set to 100 μm yielding a spectral resolution of 0.45 cm^−1^. All measurements were carried out at room temperature. The dielectric tensor as function of the wavelength and particular at the excitation wavelength was determined by means of spectroscopic ellipsometry published elsewhere^11^.

Ab-initio calculations of the phonon energies and Raman intensities were carried out using the CRYSTAL14 code. We used the basis set of Pandey *et al.*^12^ for gallium and of Valenzano *et al.*^13^ (slightly modified) for oxygen. The B3LYP hybrid functional was used as it is known to yield good agreement to experimental results for vibrational properties^13^,^14^. We could also verify this finding based on comparative calculations using other Hamiltonians (B3PW, PBE0, HSE06). Pack-Monckhorst and Gilat shrinking factors of 8 were used which corresponds to 150 *k*-points in the irreducible Brillouin zone. The truncation criteria for the Coulomb and exchange infinite sums are defined in the CRYSTAL14 code by five tolerances set to 8, 8, 8, 8, and 16 for our calculations. The tolerance for the energy convergence was set to 10^−11^ Hartree. The complete input is provided in the supplementary material. The lattice parameter optimization with these parameters yields *a*_0_ = 12.336 Å, *b*_0_ = 3.078 Å, *c*_0_ = 5.864 Å and *β* = 103.89°. This slight overestimation of the lattice parameters with respect to experimental values^15^,^16^,^17^ is expected for the B3LYP hybrid functional^13^,^14^,^18^.

## Additional Information

**How to cite this article**: Kranert, C. *et al.* Raman tensor elements of *β*-Ga_2_O_3_. *Sci. Rep.* **6**, 35964; doi: 10.1038/srep35964 (2016).

**Publisher’s note**: Springer Nature remains neutral with regard to jurisdictional claims in published maps and institutional affiliations.

## Supplementary Material

Supplementary Information

## Figures and Tables

**Table 1:** Spectral position of the Raman peaks of the phonon modes of *β*-Ga_2_O_3_, given in cm^−1^.

Phonon mode	Experiment				Theory			
	This work	ref. 4	ref. 5	ref. 6	This work	ref. 4	ref. 5	ref. 7
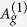	111.0	111	110.2	112	113.5	113	104	104.7
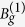	114.8	114	113.6	115	118.6	114	113	112.1
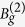	144.8	147	144.7	149	145.6	152	149	141.3
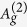	169.9	169	169.2	173	176.4	166	165	163.5
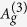	200.2	199	200.4	205	199.1	195	205	202.3
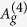	320.0	318	318.6	322	318.5	308	317	315.8
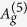	346.6	346	346.4	350	342.5	353	346	339.7
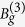	353.2	353	n.o.	355	359.2	360	356	348.3
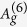	416.2	415	415.7	421	432.0	406	418	420.2
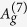	474.9	475	n.o.	479	472.8	468	467	459.4
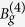	474.9	475	473.5	480	486.1	474	474	472.8
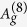	630.0	628	628.7*	635	624.4	628	600	607.1
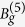	652.3	651	652.5*	659	653.9	644	626	627.1
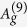	658.3	657	n.o.*	663	655.8	654	637	656.1
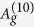	766.7	763	763.9	772	767.0	760	732	757.7

A more likely assignment for two peaks from ref. 5 indicated by “*” was chosen to enhance the comparability, “n.o.” denotes modes which were not observed.

**Table 2:** Parameters for the crystal cuts under investigation.

Facet	*θ*	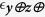	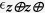	Δ*n*	(1 − *r*fast)/(1 − *r*slow)	Ωfast/Ωslow
(010)	0°	0	3.809	0.027	1.0094	1.029
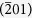	36.2°	−0.050	3.705	0.018	1.0060	1.019
(100)	−17.6°	0.031	3.678	0.011	1.0082	1.0040

The ratios of the solid angles are given for a numerical aperture of *NA* = 0.42 The slow axis (higher reflectance) is the *x*-axis for excitation on the (010)-facet and the *z*-axis for the other two facets.

**Table 3:** Raman tensor elements obtained from modeling the polarization dependence of the experimental Raman scattering intensity and from ab-initio calculations, normalized to a value of 1000 for the highest Raman polarizability.

Phonon mode	Experiment				Theory			
	*a*	*b*	*c*	*d*	*a*	*b*	*c*	*d*
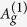	19	−59	14	13	79	−70	21	9
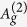	100	146	119	0	142	214	150	−34
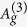	187	445	311	27	154	431	272	−16
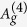	111	147	135	128	124	113	154	146
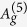	441	111	320	−5	479	12	349	−18
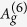	357	289	338	158	320	358	293	146
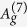	47	−300	327	−52	31	−369	−241	9
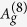	59	393	240	−135	55	414	53	−164
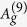	408	77	118	325	468	61	21	364
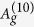	1000	353	0	−283	1000	248	−191	−409

Experimental values for *c* are given as positive values, but may as well be negative from experimental results.

**Table 4:** Raman tensor elements obtained from modelling the polarization dependence of the experimental Raman scattering intensity and from theoretical calculations, normalized to a value of 1000 for the tensor element *a* of the 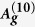 mode.

Phonon mode	Experiment		Theory	
	*e*	*f*	*e*	*f*
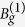	32	31	46	56
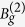	106	70	148	88
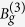	n.d.	n.d.	238	−92
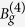	n.d.	n.d.	12	−291
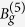	162	326	147	335

Elements marked with “n.d.” could not be determined.
