# Supplementary material for: Raman tensor elements of β-Ga2O3
Source: Sci Rep. 2016 Nov 3;6:35964. doi: 10.1038/srep35964 (PMC5093899; doi:10.1038/srep35964)
Supplement: Supplementary Information [file srep35964-s1.pdf]

Raman tensor elements of β-Ga_2O_3
–Supplementary Material–

Christian Kranert[1,2], Chris Sturm[1], Rüdiger Schmidt-Grund[1], and Marius Grundmann[1]
[1] *Universität Leipzig, Institut für Experimentelle Physik II, Abteilung Halbleiterphysik, Linnéstraße 5, 04103 Leipzig, Germany*
[2] *current address: Fraunhofer THM, Am St.-Niclas-Schacht 13, 09599 Freiberg, Germany*

1 Deviation from normal incidence due to the numerical aperture

Real Raman experiments cannot be carried out under perfect normal incidence due to the finite aperture of the focusing optics. This causes a deviation from the ideal normal incidence case which is however shown to be negligible in the following. Two aspects play a role: an out-of-plane polarization component $e_\perp$ resulting from p-polarized light and a splitting of the light into ordinary and extraordinary ray due to the birefringence. Due to the rather small birefringence of β-Ga_2O_3, the latter is only a small correction to the first effect and is therefore neglected here. Its exact description requires raytracing of the incident and scattered light within the crystal and in general cannot be described analytically. In the following, we treat the crystal as being isotropic with index of refraction $n = 1.9$.

We assume a homogeneous intensity distribution on the front element of the focusing optics and a point focus. The front element is approximated to be flat. The tilt of a single incident light ray α relative to normal incidence then depends on the radial position r from which it leaves the focusing optics by

$$\tan\alpha = \frac{d}{r} \tag{1}$$

with the focusing distance d. The maximum angle is determined by the numerical aperture NA by

$$\sin(\alpha_{\max}) = NA = \frac{R}{\sqrt{d^2+R^2}}\,. \tag{2}$$

Here, R is the diameter of the front element of the focusing optics. Since the absolute dimensions are not relevant, we can set $R = 1$ and find

$$\alpha(r) = \arctan\left(\frac{r}{\sqrt{\frac{1}{NA^2}-1}}\right)\,. \tag{3}$$

For the light propagation within the crystal, the angle changes to

$$\beta(r) = \arcsin\left(\frac{\sin(\alpha(r))}{n}\right) \tag{4}$$

due to refraction.

The azimuthal direction of the light is given by the angle ϕ which we define such that $\phi = 0$ lies in the polarization plane, or vice versa that for $\phi = 0$ the light is strictly p-polarized. The out-of-plane polarization component of an individual light ray within the crystal is then $e_\perp = \arcsin(|\sin(\beta(r))\cos(\phi)|)$. Further, the transmission at the interface is different for p- and s-polarized light which needs to be considered using the Fresnel equations. The ratio in the transmission coefficient for these two polarization states is

$$\frac{t_p}{t_s} = \frac{\cos(\alpha)+n\cos(\beta)}{n\cos(\alpha)+\cos(\beta)}\,. \tag{5}$$

Including this ratio and averaging over the area the front element of the focusing optics, we obtain

$$\bar{e}_\perp = \frac{1}{2\pi}\int_0^\pi\int_0^1 \frac{\cos(\alpha)+n\cos(\beta)}{n\cos(\alpha)+\cos(\beta)}\arcsin(|\sin(\beta)\cos(\phi)|)r\mathrm{d}r\mathrm{d}\phi\,. \tag{6}$$

The in-plane polarization component consequently is $e_\parallel = \sqrt{1-e_\perp^2}$. Since intensities are measured, the polarization enters quadratically such that the relative contribution of the out-of-plane polarization component can be written as

$$\frac{I_\perp}{I_\parallel} = \frac{\bar{e}_\perp^2}{1-\bar{e}_\perp^2}\,. \tag{7}$$

This applies to the out-of-plane component being converted into an in-plane component during the scattering event. The term for the conversion in the opposite direction is similar. If no conversion takes place, the contribution of the out-of-plane component must be squared and becomes even smaller and can thus be neglected. The result of (7) is plotted in dependence on the numerical aperture of the focusing optics in Supplementary Fig. 1. It is below 1 % for $NA < 0.7$ and can thus be neglected in good approximation. For our experimental setup with $NA = 0.42$, we obtain a value of 2.5×10^{-3}. This is in good agreement with the fact that no signs of scattering from phonon modes with B_g symmetry are observed for excitation on the (010)-plane of β-Ga_2O_3.

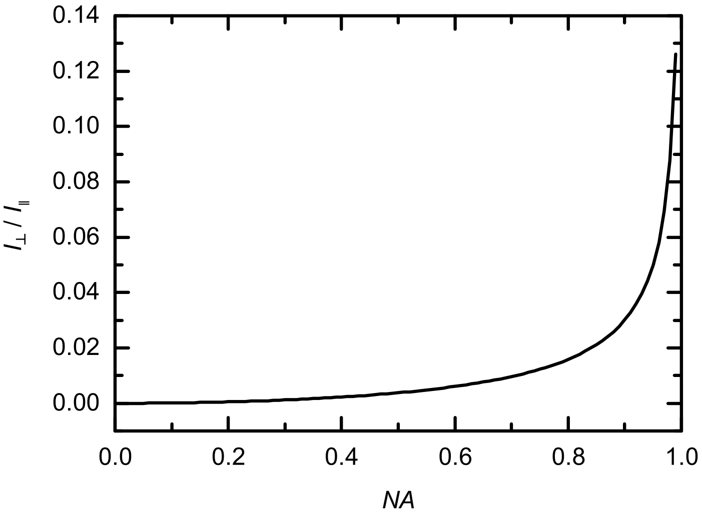

Supplementary Figure 1. Contribution of the out-of-plane polarization component to the scattering intensity relative to the contribution of the in-plane component in dependence on the numerical aperture NA.

2 Actual line shape of the depth resolution profile

We experimentally determined the line shape function for the depth resolution of our setup by tuning the focus of the microscope objective through a 1.4 µm thick film of GaN grown on sapphire substrate. We measured the Raman spectrum at each focus point and determined the intensity of the characteristic $E_2^{(2)}$ mode. This is plotted in Supplementary Fig. 2(a).

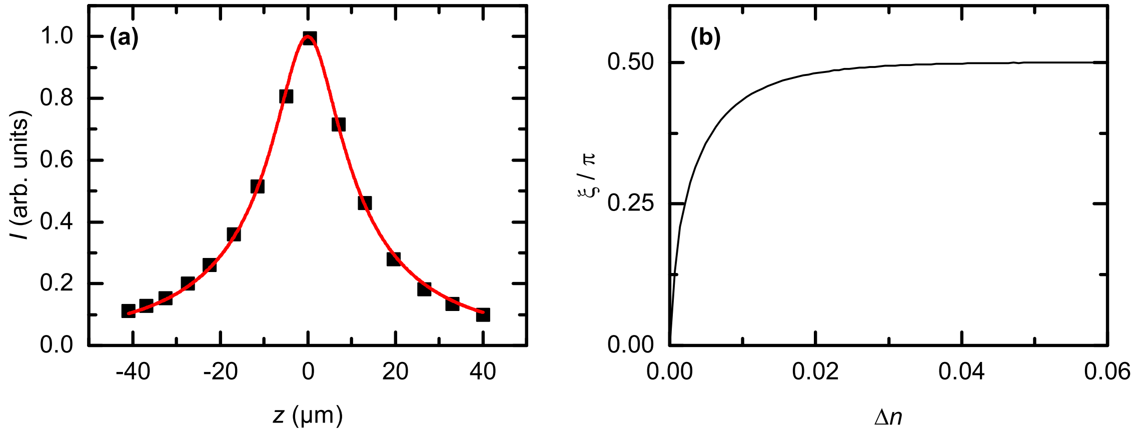

Supplementary Figure 2. (a) Experimentally determined line shape (squares) and fit (solid line) of the depth resolution profile of our experimental setup. (b) Apparent angle ξ in dependence on the magnitude of birefringence Δn for the depth line shape from (a).

The shape of the focus on the sample is determined by the entrance slit of the spectrometer which is neither a

perfect point nor line focus, but has a rectangular shape. Therefore, we used the product of two intensity profiles for two line foci with broadenings w_1 and w_2:

$$I = \frac{1}{\sqrt{1+\frac{(x-x_0)^2}{w_1^2}}} \times \frac{1}{\sqrt{1+\frac{(x-x_0)^2}{w_2^2}}}. \tag{8}$$

As can be seen from Supplementary Fig. 2, this function yields a very good agreement to the experimental data using the fitting parameters $w_1 = 7.6\,\mu m$ and $w_2 = 28.2\,\mu m$. We used this depth profile function with the experimentally determined broadenings to calculate the apparent phase angle ξ between the tensor elements of the effective Raman tensor[2]. The result is plotted in Supplementary Fig. 2 (b) in dependence on the magnitude of birefringence Δn. The values relevant for our investigations of β-Ga_2O_3 are summarized in Supplementary Tab. 1. Please note that for excitation on the ($\bar{2}01$)- and the (100)-plane of β-Ga_2O_3, only diagonal elements of the Raman tensor of the A_g modes play a role. Thus, the observed Raman intensities are determined by ξ' only[2]. The results show that for the samples investigated by us, convergence of the depth integral ($\xi = 0.5\pi$) can be assumed in good approximation. However, for an even lower Δn than for the (100) surface, the observation of depth-dependent effects is expected.

orientation	Δn	$\Delta n w/\lambda$	ξ	ξ'
(010)	0.027	0.558	0.491π	0.499π
($\bar{2}01$)	0.018	0.372	(0.477π)	0.496π
(100)	0.011	0.227	(0.442π)	0.485π

Supplementary Table 1. Parameters for the convergence of the depth integral for β-Ga_2O_3 surfaces under investigation. Values in parentheses do not affect the observed Raman scattering intensities.

3 Input for the ab-initio calculations using CRYSTAL14

```
Ga2O3 Raman intensities
CRYSTAL
0 0 0
 12
12.33573482 3.07758865 5.86360632 103.892342
5
31 .0905626 .0000 -.2054168
31 .1579964 .5000  .3141800
8  .1626020 .0000  .1097351
8  .1740538 .0000 -.4355277
8 -.0048694 .5000  .2575452
FREQCALC
INTENS
INTRAMAN
INTCPHF
ENDCPHF
ENDFREQ
END
31 8
0 0 8 2.0 1.0
 444668.  0.00023
 64576.8  0.0019
 13935.2  0.0108
 3651.66  0.0490
 1099.41  0.1672
 381.106  0.3643
 149.482  0.4028
 62.817   0.1477
0 1 6 8.0 1.0
```

```
 1155.65 -0.006  0.0088
 278.06  -0.07   0.0630
 93.1902 -0.14   0.2196
 37.0948  0.2685 0.4083
 15.4816  0.6184 0.4154
 5.1517  0.3248  0.3536
0 1 4 8.0 1.0
 70.6212  0.0067 -0.0085
 27.1784 -0.0863 -0.0350
 11.4394 -0.3455  0.0849
 4.6239   0.4086  0.5583
0 1 1 3.0 1.0
 1.8289 1.0 1.0
0 1 1 0.0 1.0
 0.66317 1.0 1.0
0 1 1 0.0 1.0
 0.240 1.0 1.0
0 3 4 10.0 1.0
 67.4273 0.0259
 18.8367 0.1495
 6.3055  0.3805
 2.1298  0.4768
0 3 1 0. 1.0
 0.66949 1.0
8 6
0 0 8 2.0 1.0
 8020.   0.00108
 1338.   0.00804
 255.4   0.05324
 69.22   0.16810
 23.90   0.35810
 9.264   0.38550
 3.851   0.14680
 1.212   0.07280
0 1 4 6. 1.0
 49.43  -0.00883 0.00958
 10.47  -0.09150 0.06960
 3.235  -0.04020 0.20650
 1.217   0.37900 0.34700
0 1 1 0. 1.0
 0.486 1. 1.
0 1 1 0. 1.0
 0.190 1. 1.
0 3 1 0. 1.
 2.0 1.
0 3 1 0. 1.
 0.500 1.
99 0
END
DFT
B3LYP
XLGRID
ENDDFT
TOLINTEG
8 8 8 8 16
```

```
SHRINK
8 8
TOLDEE
11
MAXCYCLE
150
END
```

References

1. Papagiakoumou, E., de Sars, V., Oron, D. & Emiliani, V. Patterned two-photon illumination by spatiotemporal shaping of ultrashort pulses. *Optic Express* **16**, 22039–22047 (2008).
2. Kranert, C., Sturm, C., Schmidt-Grund, R. & Grundmann, M. Raman Tensor Formalism for Optically Anisotropic Crystals. *Phys. Rev. Lett.* **116**, 127401 (2016).
